# MicroRNAs与*OCT4*基因之间的相互作用

**DOI:** 10.3779/j.issn.1009-3419.2015.01.09

**Published:** 2015-01-20

**Authors:** 琛 陈, 海粟 万

**Affiliations:** 300052 天津，天津医科大学总医院，天津市肺癌研究所，天津市肺癌转移与肿瘤微环境实验室 Tianjin Key Laboratory of Lung Cancer Metastasis and Tumor Microenviroment, Tianjin Lung Cancer Institute, Tianjin Medical University General Hospital, Tianjin 300052, China

**Keywords:** MicroRNAs, OCT4, 肿瘤, 胚胎干细胞, 多能性, MicroRNAs, OCT4, Cancer, Embryonic stem cell, Pluripotency

## Abstract

*OCT4*基因是POU转录因子家族中的一员，它能与含八聚体基序（ATGCAAAT）的DNA结合。OCT4是一个关键的转录因子，在未分化胚胎干细胞中参与维持多能性和自我更新性，在许多种癌症包括肺癌、生殖细胞肿瘤、乳腺癌、宫颈癌、前列腺癌、胃癌、肝癌和卵巢癌中过表达。MicroRNAs（miRNAs）是一种小的非编码RNA，通过和靶基因mRNA碱基配对来调控mRNA表达，降解mRNA或阻碍蛋白合成。一些miRNAs被证实在癌细胞中调控干细胞因子如OCT4、NANOG、SOX2和KLF4，进而调控癌细胞的增殖、凋亡、分化、抗药性和免疫性。

## Abstract (en)

Octamer-binding transcription factor 4 (OCT4) belongs to the POU-homeodomain family of transcription factors and binds to an octamer motif, ATGCAAAT. OCT4 is the key transcription factor that is involved in the maintenance of pluripotency and self-renewal in undifferentiated embryonic stem (ES) cells. OCT4 has been reported to be overexpressed in various cancers including lung, germ cell tumors, breast, cervix, prostate, gastric, liver, and ovarian cancer. MicroRNAs (miRNAs), small non-coding RNAs, modulate mRNA expression through base pairing between seed sequences in miRNA and complementary sequences of the target mRNA, thereby destabilizing mRNA and/or inhibiting protein synthesis. Several miRNAs have been demonstrated to regulate stemness factors such as OCT4, NANOG, SOX2 and KLF4 in cancer cells, thereby modulating the proliferation, apoptosis, differentiation, drug resistance and immunity of cancer cells.

## 1 OCT4介绍

哺乳动物在胚胎发育过程中受着多种基因的严密调控，以保证其有序地进行组织分化和个体发育。*OCT4*基因就是其中一个关键基因，也被称为OCT3、POU5F1、OTF3或OTF4，是POU转录因子家族中的一员^[1, 2]^。人的*OCT4*基因位于6号染色体上（6p21.31），长度为16.40 kb，具有多个转录起始位点，转录不同的mRNA亚型（Isoform），从而翻译成多种蛋白质^[3]^。OCT4 Isoform 1是转录的主要亚型之一，具有5个外显子、4个内含子，翻译的蛋白质含有一个保守的DNA结合结构域——POU结合域，它能与含八聚体基序（octamer motif）的DNA结合从而调控下游靶基因的转录^[4]^。OCT4是一个关键的转录因子，在未分化胚胎干细胞中参与维持多能性和自我更新性^[5]^，在许多种癌症包括肺癌^[6]^、生殖细胞肿瘤^[7]^、乳腺癌^[8]^、宫颈癌^[9]^、前列腺癌^[10]^、胃癌^[11]^、肝癌^[12]^和卵巢癌^[13]^中过表达。

## 2 MicroRNAs（miRNAs）介绍

miRNAs是一种小的、类似于siRNA的分子，由高等真核生物基因组编码，miRNA通过和靶基因mRNA碱基配对引导沉默复合体（RNA-induced silencing complex, RISC）降解mRNA或阻碍其翻译^[14]^。miRNAs在物种进化中相当保守，在植物、动物和真菌中发现的miRNAs只在特定的组织和发育阶段表达，miRNAs组织特异性和时序性，决定组织和细胞的功能特异性，表明miRNAs在细胞生长和发育过程的调节过程中起多种作用^[15]^。目前，在哺乳动物基因组中，已鉴定出上百种miRNAs，但只有小部分明确了其功能特点^[16]^。

miRNAs基因通常是在核内由RNA聚合酶Ⅱ（polⅡ）转录的，最初产物为大的具有帽子结构（7MGpppG）和多聚腺苷酸尾巴（AAAAA）的pre-miRNA^[17]^。miRNAs在核酸酶Drosha和其辅助因子Pasha的作用下被处理成70个核苷酸组成的pre-miRNA。RAN-GTP和exportin 5将pre-miRNA输送到细胞质中。随后，另一个核酸酶Dicer将其剪切产生约为22个核苷酸长度的miRNA:miRNA^*^双链。这种双链很快被引导进入RISC中，其中一条成熟的单链miRNAs保留在这一复合体中^[18]^。miRNAs结合到与其互补的mRNA的位点通过碱基配对调控基因表达^[19]^。

## 3 与OCT4相关的miRNAs

在细胞中，许多miRNAs都与*OCT4*基因相关，如miR-145、miR-302、miR-125b、miR-430、miR-200、miR-335。而且OCT4与这些miRNAs之间的相互影响对肿瘤细胞起到调节作用。

### 3.1 miR-145

miR-145通过结合到转录因子OCT4、SOX2和KLF4的3’UTR来调控这些因子。这种调控作用在许多肿瘤中都存在，如胸膜间皮瘤^[20]^、肝癌^[21]^、胰腺癌^[22]^、非小细胞肺癌^[23]^和前列腺癌^[24]^。

在肺腺癌中，miR-145的表达水平下调，和OCT4表达水平负相关。miR-145作为肿瘤抑制剂通过调控OCT4表达来下调肺癌起始细胞的肿瘤干细胞特性和上皮细胞间质转型（epithelial-mesenchymal transition, EMT），抑制肿瘤生长和转移^[25]^。

Chinnathambi等^[26]^研究发现，在胚胎干细胞（embryonic stem cell, ESCs）中存在一个OCT4和miR-145之间的平衡，在人胚胎干细胞（human embryonic stem cell, hESCs）中，OCT4高表达，而miR-145表达低。当miR-145在hESCs中升高时，多能性重编程因子OCT4、SOX2、KLF的未翻译区被抑制。此外，OCT4能够结合并抑制miR-145的启动子。因此，OCT4和miR-145之间互相调控。

OCT4和miR-145之间的平衡会被多种因素调控。例如：缺氧条件可以调控Oct4/miR-145平衡进而调整人肾髓质CD133前体细胞的未分化表型^[27]^。姜黄素通过上调miR-145来降低OCT4A、OCT4B1、SOX2和NANOG的表达，进而减少U87MG细胞的增殖^[28]^。

Wang等^[29]^研究发现，OCT4的一个假基因OCT4-pg4，在肝癌细胞中被激活。OCT4-pg4的表达水平与OCT4正相关，这两种基因转录都被抑制肿瘤的miR-145作用。OCT4-pg4有生物活性，可以保护OCT4转录不被miR-145抑制，上调OCT4蛋白在肝细胞癌（hepatocellular carcinoma, HCC）中的水平。OCT4-pg4可以促进HCC的生长，因此，在肝癌中起到致瘤性的作用。

### 3.2 miR-302

miR-302基因编码8个miRNA的miRNAs簇（miR-302b^*^-b-c^*^-c-a^*^-a-d-367），这几个miRNAs簇在hESCs和肿瘤胚胎细胞中表达，提高细胞重编程效率^[30]^。

在发育的早期阶段，OCT4和miR-302在同一个组织同时表达。OCT4、SOX2和NANOG结合到miR-302的启动子，活化miR-302在hESCs中的转录^[31]^。当miR-302a在非多能性细胞中表达时，抑制了cyclin D1的mRNA，导致cyclin D1蛋白水平降低，丧失G_1_/S checkpoint，调控细胞从G_1_期到S期转换，S期细胞升高，G_1_期细胞降低。相反，抑制hESCs中的miR-302时会导致G_1_细胞数量增加^[32]^。在HeLa细胞、成纤维细胞和皮肤癌细胞中，表达外源miR-302可以诱导细胞快速增殖，提高S期细胞数，降低G_1_期细胞数^[26]^。

Brautigam等^[33]^对比了miR-290簇和miR-302簇作用的区别，miR-290簇miRNAs在鼠胚胎的4个细胞期上调，在胚囊期达到最高水平，而miR-302簇miRNAs在E6.5开始表达，在E7.5时达到峰值。这些簇的特定表达模式表明两种簇的miRNAs在胚胎发育的不同阶段很重要。miR-290簇miRNAs在mESCs中和体内胚囊期有重要作用，而miR-302簇miRNAs在后面阶段有重要作用。

### 3.3 miR-125b

作为最早发现的miRNAs之一，miR-125b是主要的致癌因子，参与增殖、凋亡、分化、耐药性和免疫。miR-125b作为原癌miRNAs，通过抑制凋亡来促进肿瘤生长^[34, 35]^。

利用染色质免疫沉淀分析发现，在宫颈癌和畸胎癌细胞中，OCT4结合在miR-125b-1的启动子上，上调miR-125b的表达。miR-125b的过表达能够抑制凋亡和BAK1的表达。Western blot分析表明，在宫颈癌细胞中BAK1表达与miR-125b表达负相关，与OCT4水平不相关。Luciferase数据也证实了BAK1是miR-125b的直接的靶基因，OCT4通过miR-125b来抑制BAK1表达。这说明OCT4间接作用到BAK1诱导其下调，进而抑制细胞凋亡。IHC分析表明，BAK1的表达在原发宫颈癌中与OCT4负相关，而miR-125b与OCT4正相关，进一步证实OCT4的通过活化miRNA-125b/BAK1通路导致宫颈致癌^[36]^。

### 3.4 miR-430

miR-430是目前已发现的最大miRNAs基因簇，在斑马鱼和非洲爪蟾中起到清除母系mRNA的作用，它被NANOG、POU5f1和SOXB1共同调控。miR-430负责在母型-合子型过渡（maternal-to-zygotic transition, MZT）过程清除母系的mRNA，促进向合子转化。保守的miR-430/295/302/372家族的miRNAs稳定了胚胎干细胞的自行更新，加强了重编效率^[37]^。这些miRNAs通过加速去除前面程序的mRNA来消除记录，通过重编因子促进建立新的转变状态。NANOG、SOXB1和POU5f1导致miR-430表达上调进而提供了启动合子基因激活和清除之前母系记录之间的联系^[38]^。

### 3.5 miR-200

miR-200家族包括5个成员：miR-200a、miR-200b、miR-200c、miR-141和miR-429。这些miRNAs在调控肿瘤细胞的增殖、自我更新、分化中起重要作用。如miR-200b可以抑制前列腺癌的细胞增殖、迁移以及提高化疗敏感度^[39]^。

miR-200家族5个成员定位在小鼠的2个染色体上的2个簇（染色体4：miR-200a/b/429；染色体6: miR-141/200c）。OCT4和SOX2能结合到miR-141/200c和miR-200a/b/429各自的启动子，激活miR-200s的转录。研究发现OSKM诱导多能干细胞（induced pluripotent stem cells, iPSC）的繁殖需要miR-200，结合这一点可以看出，内源miR-200s作为OCT4和SOX2唯一的介质来诱导细胞重编程。

在iPSC繁殖的早期阶段，miR-200/ZEB2通路的激活是OCT4/SOX2的一个重要功能。OCT4和SOX2通过结合到miR-200家族的启动子区域直接激活miR-200家族的表达，而miR-200s的表达会作用到ZEB2的3′ UTR进而抑制ZEB2的表达。miR-200/ZEB2通路帮助成纤维细胞越过MET屏障，促进iPSC繁殖^[40]^。

### 3.6 miR-335

Schoeftner等^[41]^研究发现了一个连接miR-335、OCT4、视网膜母细胞瘤的调控通路，这个通路控制mESCs的自我更新和分化。OCT4驱使Nipp1和Ccnf表达，抑制磷酸酶1（phosphatase 1, PP1）蛋白复合物的活性，建立视网膜母细胞瘤蛋白质（retinoblastoma protein, pRb）磷酸化，而pRb磷酸化是mESCs自我更新的标志性特征。miR-335调控OCT4和Rb的表达，在miR-335控制下，OCT4-Nipp1/Ccnf-PP1-pRb轴促进mESCs自我更新。当mESCs分化时，miR-335上调，OCT4转录抑制，使Oct4-Nipp1/Ccnf-PP1-pRb轴崩溃，pRb去磷酸化，退出自我更新，建立了一个pRb调控细胞周期的程序。

Gao等^[42]^发现，OCT4是miR-335的一个直接的功能目标，miR-335通过作用OCT4来抑制胰腺癌细胞的增殖和干细胞特性。系统地输送miR-335可以抑制胰腺癌转移，延长生存。

## 4 总结

*OCT4*基因是维持胚胎干细胞多潜能性和自我更新的最关键基因，在许多肿瘤中高表达，许多种miRNAs通过与OCT4作用来调控肿瘤的生长、转移以及化疗敏感度。这些发现可能将会为肿瘤治疗提供新的手段。
